# Natriuretic Peptides as a Predictor for Delirium After Cardiac Surgery: A Prospective Observational Study

**DOI:** 10.3390/jcm14051533

**Published:** 2025-02-25

**Authors:** Cyril Huisinga, Eric Struck, Lorenz Mihatsch, Jens Heyn, Christian Hagl, Bernhard Zwissler, Vera von Dossow, Thomas Saller

**Affiliations:** 1 Department of Anaesthesiology, LMU University Hospital, Ludwig-Maximilians-Universität, 81377 Munich, Germany; ericstruck1610@gmx.de (E.S.); jens.heyn@web.de (J.H.); bernhard.zwissler@med.uni-muenchen.de (B.Z.); tsaller@med.lmu.de (T.S.); 2 Technical University of Munich, TUM School of Medicine and Health, Children’s Hospital, 80803 Munich, Germany; l.mihatsch@tum.de; 3 Department of Cardiac Surgery, LMU University Hospital, Ludwig-Maximilians-Universität, 81377 Munich, Germany; christian.hagl@med.uni-muenchen.de; 4 Institute for Anesthesiology, Heart and Diabetes Center NRW, Ruhr University of Bochum, 44801 Bad Oeynhausen, Germany; vvondossow@hdz-nrw.de

**Keywords:** delirium, neurocognitive disorders, biomarker, natriuretic peptides, cardiac surgery, short form 36 questionnaire, quality of life

## Abstract

**Background/Objectives**: Subsequent to surgical procedures under cardiopulmonary bypass, at least one third of the patients experience delirium. Among others, the disruption of the blood–brain barrier results from the release of natriuretic peptides during surgery. Furthermore, natriuretic peptides increase the effect of dopamine agonists, which is a key element in the pathomechanism of delirium. The primary endpoint of this study was the adjusted mean difference in natriuretic peptide concentration before surgery between patients with and without delirium. The secondary endpoints were the differences in cognitive performance and quality of life, and physical performance. **Methods**: Single center observational study. Setting in the Cardiac surgery and intensive care at a German tertiary medical center. Eighty patients for elective cardiac surgery under cardiopulmonary bypass for valve replacement or coronary artery bypass grafting. Preoperative NT-pro C-type natriuretic peptide (CNP) was determined. After surgery, delirium was assessed five times daily using the confusion assessment method for intensive care until 72 h after surgery and before hospital discharge. Data on quality of life and physical performance were also collected. **Results**: Overall, 28/80 (35%) patients developed delirium. Patients with delirium showed an increased concentration of NT-proCNP preoperatively (*p* = 0.016) compared to those who did not experience delirium. Patients with delirium during hospitalization reported deterioration in their physical role function (*p* = 0.036), vitality (*p* = 0.004), and social function (*p* = 0.008) before surgery. **Conclusions**: Increased NT-proCNP before surgery is associated with the occurrence of delirium. A relevant reduction in cognitive and physical performance and quality of life may be a new risk factor for delirium.

## 1. Introduction

Demographic change is characterized by an increasing and aging population accompanied by functional and cognitive decline [1]. Besides patients’ age [2], postoperative neurocognitive disorders (NCDs) contribute to cognitive impairment in older people. NCDs have been described in 53% of patients after coronary bypass surgery at discharge, 36% at six weeks after discharge, and in 24% [3] up to 43% [4] at six months after discharge.

Patients who develop delirium have a significantly increased risk of death during hospitalization or prolonged hospitalization, and experience a significant and long-term reduction in cognitive performance [5]. In cardiac surgery, delirium is the strongest predictor for re-intubation and the need for tracheostomy as well as a longer length of stay in the intensive care unit (ICU) and hospital in general [6].

The probability of death in the first three months increases by 11% with each 48 h duration of active delirium [7]. Additionally, delirium is misdiagnosed, diagnosed with delay or even too late, or not recognized at all in more than 50% of cases. [8,9]. The scores to calculate the risk of delirium in cardiac surgery mostly show poor performance in their ability to predict delirium [10,11,12].

The exact mechanisms of delirium are still the subject of current research. There are several hypotheses regarding the pathogenesis of delirium.

Inflammation and neuroinflammation appear to be a crucial mechanism in the development of postoperative delirium. Surgical procedures trigger a systemic inflammatory response that leads to the release of pro-inflammatory cytokines (interleukin-1/6, tumor necrosis factor-alpha (TNF-α)). These cytokines enter the central nervous system (CNS) and activate microglia. This releases further inflammatory mediators and intensifies neuroinflammation [13,14,15]. Inflammatory markers are relevant for delirium [16,17]. However, they are unspecific, like the C-reactive protein (CRP) [17,18]. Since inflammation is common and only a limited number of patients with inflammation develop delirium, biomarkers for blood–brain barrier and astrocyte damage such as S100B could be useful [19]. However, all these biomarkers were developed for other indications.

Endothelial dysfunction due to damage to the blood–brain barrier is associated with severe inflammation, vasoplegia, and delirium [20]. The inflammatory response increases the permeability of the blood–brain barrier. Normally, the blood–brain barrier has the function of protecting the brain from harmful substances. The cytokines described can lead to damage of the tight junctions between the endothelial cells. This allows neurotoxic substances, immune cells, and cytokines to enter the CNS and contribute to the development of POD. Inflammatory mediators in the brain can impair synaptic plasticity and neurotransmission, thus further exacerbating cognitive dysfunction [13,14,15]. Endothelial dysfunction in cardiac surgery patients is caused, among other things, by the release of natriuretic peptides (atrial natriuretic peptide, ANP, and C-type natriuretic peptide, CNP) [21]. In addition to damaging the endothelial glycocalyx, ANP appears to enhance the effect of dopamine agonists and thus play a possible role in the pathomechanism [22] and increase the development of delirium. So far, natriuretic peptides have not been determined in patients who develop postoperative delirium.

An imbalance in neurotransmission also appears to play a role, in particular an excess of dopamine and a deficiency in acetylcholine. Increased dopamine can lead to hyperactivity and agitation. A deficiency in dopamine can promote attention deficits. Acetylcholine plays a crucial role in learning, attention, and memory. A deficiency in acetylcholine contributes to cognitive impairment. Anesthesia and stress can contribute to acetylcholine deficiency [13,14,15].

Another key element is oxidative stress and mitochondrial dysfunction. Surgical interventions can favor the production of reactive oxygen species. These are by-products of cell metabolism that can lead to neuronal damage, particularly to lipids, proteins, and DNA. Oxidative stress is associated with neuroinflammation, which intensifies the pathological mechanism described above [15].

Biomarkers increasingly became the focus of delirium research. In addition to their role as surrogate parameters for delirium, they could also enable risk assessment and monitoring of delirium in the future [13,23].

Unfortunately, ANP is unstable and not suitable for preoperative assessment in routine clinical practice. In contrast, the NT pro-peptide of ANP is stable enough to be detected in blood drawn for preoperative assessment before cardiac surgery. The same is true for CNP, although it is a paracrine and autocrine protein and is the most active natriuretic peptide in the human brain [17]. Yet, the physiological role of CNP is unclear, although its receptor, NPR-C is highly expressed in the brain. NT-proCNP is a more stable precursor of CNP that predominates in the brain and is secreted in equimolar amounts. With normal renal function, NT-proCNP accurately reflects the total amount of CNP produced, whereas CNP is rapidly degraded [24,25]. We did not analyze the brain natriuretic peptide (BNP), as it is the least specific for delirium. The most important stimulus for peptide synthesis and secretion is myocyte stretch. Therefore, BNP serves as a marker for heart failure [26].

We hypothesize that preoperative levels of NT-proCNP differ between patients with and without postoperative delirium. Therefore, this study addresses the question of whether NT-proCNP or NT-pro ANP are possible preoperative biomarkers for predicting delirium after cardiac surgery.

## 2. Material and Methods

### 2.1. Study Design and Patient Population

The ethics committee of the Faculty of Medicine, Ludwig-Maximilians-Universität (LMU), Munich, approved the study on 22 December 2016 (716-16). The vote of the Ethics Committee was carried out as follows: positive vote/ favorable opinion. Between March 2017 and October 2018, patients were screened for eligibility and written informed consent was obtained prior to inclusion. The study was prospectively registered in the German Clinical Trial Register (DRKS00011833) and adheres to the STROBE statement for prospective observational cohort studies.

### 2.2. Inclusion and Exclusion Criteria

**Figure 1:**
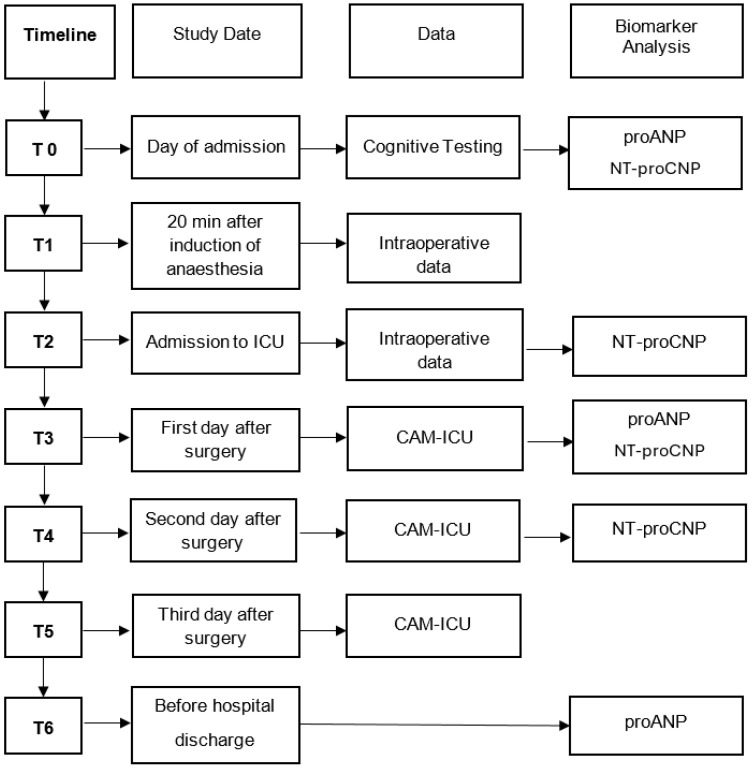
Depicts the workflow of the study. The study was structured in time points (T0 to T6). T0 describes the day of hospital admission, T1 the time 20 min after induction of anesthesia, T2 the time of admission to the ICU after surgery, T3 the first postoperative day, T4 the second postoperative day, T5 the third postoperative day, and T6 that of discharge from hospital. Confusion assessment method for intensive care (CAM-ICU).

**Figure 2:**
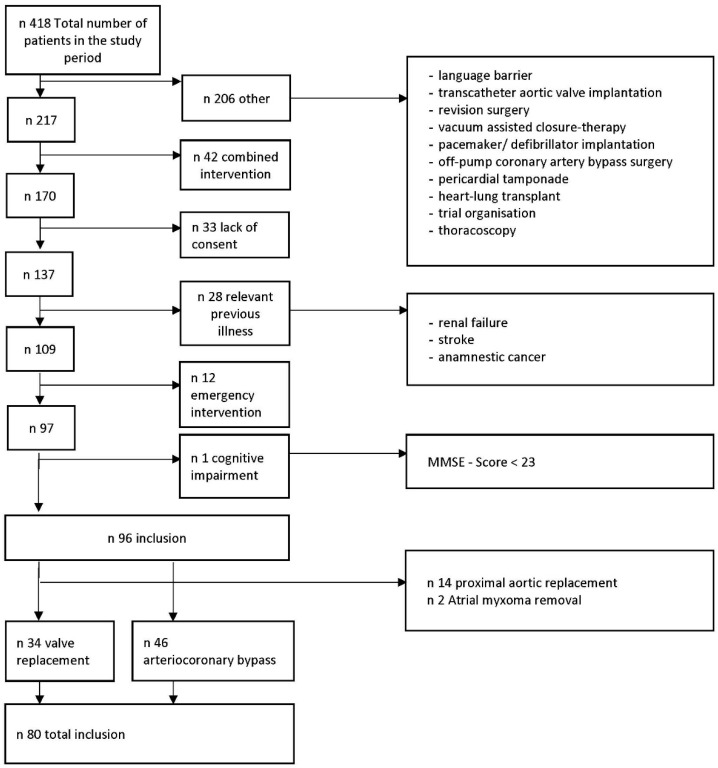
Gives an overview of the patients screened with the respective inclusion and exclusion criteria.

All patients scheduled for cardiac surgery at our hospital were considered as potential participants in our trial (see Figure 1 and Figure 2). We followed this procedure to avoid any selection bias or missing to enroll an eligible patient. Due to the high number of emergency and/or combined cardiac surgical procedures, this resulted in a relatively high number of patients being not eligible for our study due to the exclusion criteria. Enrolled patients underwent elective arterio-coronary bypass (N = 46) or heart valve replacement (N = 34) at the LMU University Hospital, LMU, Munich. All patients were perfused via cardiopulmonary bypass (CPB) during the procedure. The exclusion criteria for the study were the following:

- Age < 60 years;
- Combined aortic surgery;
- Emergency surgery;
- Renal insufficiency (glomerular filtration rate (GFR) < 60 mL/min);
- Liver cirrhosis with hepatic encephalopathy;
- Pulmonary hypertension;
- Psychiatric illness (severe dementia, schizophrenia, or depression);
- Severe preoperative cognitive impairment.

### 2.3. Cognitive Testing and General Patient-Specific Characteristics

To exclude severe preoperative cognitive impairment, a Mini-Mental Status Examination (MMSE) was performed, which required a minimum score of 23 out of 30 points for inclusion. The MMSE is a tool for the detection of dementia in clinically untested individuals aged 65 years and older in general and primary care. The cutoff value set for the MMSE defines a cognitive function that is described as normal and without limitations. This is usually set at 24, although values between 1 and 30 are theoretically possible [27].

As a further neurocognitive assessment, the Trail-Making Tests (TMT) A and B were performed before surgery. As we are planning a long-term follow-up analysis, the specific results of the cognitive tests are not presented in detail. In order to rule out dementia and quantify cognitive impairment, we demonstrate an MMSE in this manuscript.

### 2.4. Comorbidity

Pre-existing conditions, especially cardiovascular disease, were recorded. The Charlson Comorbidity Index determines 10-year mortality of chronic morbidity. A total of 13 organ systems, depending on disease severity and patient age, were included [28,29]. In addition, concomitant medication was recorded, and the anticholinergic drug scale (ADS) was determined to estimate anticholinergic burden [30].

The CAGE questionnaire was used to test patients’ alcohol drinking habits. The acronym CAGE is made up of the following subjects which are answered in binary form with Yes (1 point) or No (0 points) in each of the four categories: Cut down drinking (“Have you ever felt you ought to cut down on your drinking?”), Annoying (“Have people annoyed you by criticizing your drinking?”), Guilty (“Have you ever felt or bad or guilty about your drinking?”), and Eye opener (“Have you ever had a drink first thing in the morning to steady your nerves or get rid of a hangover?”). The recommended threshold for the CAGE test is ≥2 to detect alcohol abuse or dependence [31].

Finally, the education level (with completion of secondary school, high school, and university) and professional career of the study participants were recorded.

### 2.5. Health-Related Quality of Life, Depression, and Activity

To assess the general physical activity and vitality of patients and screen for depressive symptoms, a collection of questionnaires was completed independently by the patients the day before surgery, namely the Short Form 36 Health Survey Questionnaire (SF-36) [32], Activities of Daily Living (ADL), and Beck Depression Inventory-II (BDI).

The SF-36 is a questionnaire for assessing health-related quality of life. It consists of 36 questions to be answered subjectively by the patient. There are questions with binary response options as well as some questions with multiple (up to six) response options. The respective answer choices result in different point values on a scale. The 36 different answer choices (items) are distributed over eight different scales (see Supplementary Table S1). Different combinations of items are assigned to each scale, and the average of the scores is calculated. The higher the score, the better the patient’s quality of life. Each scale is used to assess one dimension of subjective health perception. Patients received the questionnaire on the day of their admission to the clinic and thus had sufficient time to complete it themselves at their leisure.

For the statistical evaluation of the SF-36, the means and standard deviations of a comparative study with 2471 patients were first calculated. Subsequently, the z-score of the respective dimensions could be calculated based on this reference value [33], which was used to analyze the study data.

The ADL scale has different characteristics, which are divided into five dimensions: Personal Care, Transfer, Household Activities, Money Management, and Medical Care. Here, the overall performance is assessed in dressing, bathing, eating, going to bed or getting up, using the toilet, and controlling urination and defecation. The individual items can be answered in a four-point Likert-scale. Subsequently, an index can be determined that provides information about physical activity and independence in everyday situations [34,35].

The Beck Depression Inventory-II (BDI) consists of 21 groups of statements describing typical depression symptoms. The patient subjectively ticks one of four expressions. The evaluation is conducted by summing up the individual sum values. The sum values express the severity of the depression.

### 2.6. Conduct of Anesthesia and Surgery

When non-invasive and invasive monitoring through an arterial line was in place, anesthesia was induced with a single dose of sufentanil (1 μg kg^−1^ body weight), propofol (1 mg kg^−1^ body weight) and rocuronium (0.5–1 mg kg^−1^ body weight). After intubation, an 8.5 French 4 lumen central venous line and an 8.5 French sheath were established.

During surgery, a continuous infusion of sufentanil (1 μg kg^−1^ body weight h^−1^) and propofol (4–6 mg kg^−1^ body weight h^−1^), together with a low dose of sevoflurane (0.5–0.8 Vol%), was used for the maintenance of anesthesia.

### 2.7. Extraction and Measurement of Natriuretic Peptides

In all study participants, 9 mL of whole blood was collected from a peripheral vein into ethylenediaminetetraacetic acid (EDTA) and serum containers (Monovette Sarstedt, Nümbecht, Germany). Blood samples were drawn on the day of admission, 20 min after the induction of anesthesia, on admission to the intensive care unit (ICU) immediately after surgery, and each morning during the first 72 h at the ICU or intermediate care unit (IMC). After surgery, blood samples were usually collected via a central venous catheter. After a 30 min rest period, samples were immediately centrifuged for 10 min at a speed of 2.500 rpm (EBA 20, Hettich Centrifuges, Tuttlingen, Germany). The serum and plasma were transferred to opaque 1.5 mL polypropylene tubes and immediately frozen at −80 °C until analysis. Please see Figure 1 for an overview of the study assessments performed.

The preoperative values of natriuretic peptides were determined by an enzyme-linked immunosorbent assay (ELISA), as described previously [23]. For the detection of NT-proCNP and proANP, we used an ELISA kit (Biomedica, Vienna, Austria) with a lower detection limit of 0.2 pmol/L.

Both natriuretic peptides were analyzed on the day of admission to the hospital (corresponds to preoperatively), after the induction of anesthesia, on admission to the intensive care unit, and in the first 72 h after surgery. For the 2nd and 3rd postoperative days, the analysis focused on the data for NT-proCNP but not NT-proANP, as preliminary data showed ANP not to be valid postoperatively.

### 2.8. Detection of Delirium

During the first three days after surgery, delirium was assessed daily using the Confusion Assessment Method for the Intensive Care Unit (CAM-ICU) during rounds at the ICU or IMC. Various levels are recorded in the CAM-ICU. The first step is to check whether there are any psychological changes (acute onset or fluctuating behavior over the course of the day). If this is the case, attention is tested and the patient must actively participate. For example, with the word ANANASBAUM, the hands must be pressed with each A. For ≥3 errors, changes in consciousness are recorded using the Richmond Agitation–Sedation Scale (aggressive +4, strongly agitated +3, agitated +2, restless +1, alert and calm 0, drowsy −1, lightly sedated −2, moderately sedated −3, deeply sedated −4, unable to awaken −5). Finally, disorganized thinking is tested. For this, the patient is asked logical and illogical questions, which must be answered correctly or incorrectly. According to the CAM ICU, delirium is present if the first two points mentioned above and an additional point (altered consciousness or disorganized thinking) are present. We use the CAM-ICU because it is considered one of the most sensitive and specific tests as an assessment tool for screening delirium in critically ill patients [36]. Assessments were carried out five to six times a day, twice by the study team and three times by the nursing staff in the intensive care unit. If there were any relevant differences between the study team and nursing staff, a further assessment was carried out by the study director. The assessments ran for 72 h. However, if a clear delirium developed during the course of the study, these data were also included in the evaluation.

### 2.9. Intraoperative Data

Intraoperative data were taken from the electronic anesthesia record (NarcoData, Imeso GmbH, Gießen, Germany). Vital signs (mean arterial blood pressure, temperature, blood loss), medication (sedatives, opiates, catecholamines, crystalloid infusions, transfusions, steroids), and surgical-specific data (duration of the procedure, cardiopulmonary bypass time (CPB time), aortic clamping time, reperfusion time, balance of the cardiopulmonary machine) were recorded.

### 2.10. Statistical Analysis

To evaluate the predictive ability of NT-proCNP for postoperative delirium, we performed a receiver operating characteristic (ROC) analysis. The ROC curve was constructed by plotting the true positive rate (sensitivity) against the false positive rate (1 − specificity) at various NT-proCNP thresholds.

The optimal cutoff value was determined using Youden’s index, calculated as follows:

J = Sensitivity + Specificity − 1

The threshold that maximized this index was selected as the optimal cutoff for NT-proCNP. To assess the overall discriminatory ability of NT-proCNP, we computed the area under the curve (AUC). The sensitivity and specificity at the optimal cutoff were reported. All statistical analyses were conducted using the pROC-package, version 1.18.5.

Patients were divided into two groups: those with occurrence of delirium in the first 72 h after surgery and those without. Continuous variables were tested using the Mann–Whitney U test, and for categorical variables, the χ^2^-test was used. To adjust for sex, age, and renal function, we used the least squares mean estimates derived from linear regression models for the primary and secondary endpoints. Bonferroni correction was applied to the primary endpoints to account for multiple testing. A two-sided *p* < 0.05 was considered a statistically significant result.

A sample size calculation was performed by a statistician from the Institute of Medical Information Processing, Biometry, and Epidemiology (IBE), Ludwig-Maximilians-Universität, Munich, Germany, using the tool G*Power, version 3.1.9.2 (Heinrich-Heine-Universität, Düsseldorf, Germany) [37] based on the mean values of NT-proANP between the study groups, as determined in the preliminary work by Bruegger and Schwartz [18], which were 7.1 and 9.4 ng/g (SD 0.85 and 2.89). The calculation was designed for the “worst-case” scenario, assuming that only 30% of the patients are affected by the complication of delirium. A level of significance of α = 0.05 and a power of (1 − β) = 0.95 was stipulated.

Under these assumptions and requirements, a t-test would indicate a sample size of N = 86 (power 0.953). However, given the highly divergent standard deviations, the Satterthwaite–Welch test was used with SAS PROC POWER, version 9.2 (SAS Institute, Cary, NC, USA), resulting in a required sample size of N = 80 (power 0.957), upon which we planned our study.

IBM SPSS Statistics 24 (IBM Corp., Armonk, NY, USA) and R, version 4.1.1 (“Funny Looking Kid”, R Foundation for Statistical Computing, Vienna, Austria), were used for statistical analysis of the data. Microsoft Excel (Microsoft Corp., Redmond, WA, USA) was also used to create tables and graphs.

## 3. Results

A total of 416 patients were screened. Of these patients, 80 met the criteria for participation in the study and were included (Figure 2). Demographic factors did not significantly differ between patients who developed delirium and those who did not. Table 1 shows the baseline characteristics in all patients and in the respective groups, with and without the occurrence of delirium. The physical (visual and hearing aids, falls, timed up and go, Charlson Comorbidity score, Euroscore, ejection fraction, arteriosclerosis), nursing (ADL), and mental status (BDI) of the study participants before surgery was comparable. However, visual disturbance was more frequently observed in patients with later delirium in association with the use of glasses (*p* = 0.035) and the occurrence of falls (*p* = 0.047) within the previous 12 months.

**Figure 3:**
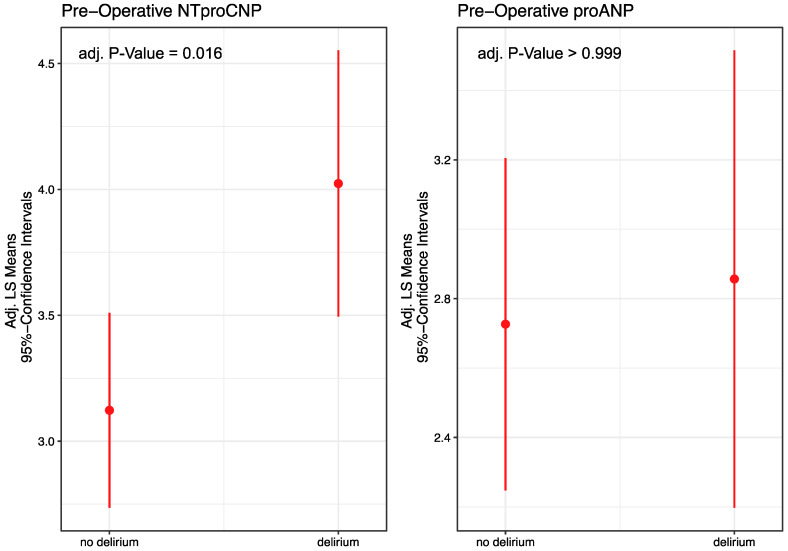
Adjusted least squares mean estimates and the respective 95% confidence intervals for the two primary endpoints: pre-operative NT-proCNP and proANP for patients with and without delirium.

The primary hypothesis of this study was to analyze natriuretic peptides as potential pre-operative biomarkers for delirium after cardiac surgery. For this purpose, the natriuretic peptides NT-proANP and NT-proCNP were determined as surrogates of endothelial activation and brain–blood barrier damage. To assess the predictive value of NT-proCNP for postoperative delirium, we performed an ROC analysis. The optimal cutoff value of 2.834 pmol/L, determined by the Youden’s index, showed a sensitivity of 75.0% and specificity of 48.1%. The AUC was 0.65, indicating moderate discrimination ability (Supplementary Figure S1). Preoperatively elevated levels of NT-proCNP (adj. *p* = 0.016) but not NT-proANP in serum (adj. *p* > 0.999) were associated with delirium adjusted for age, sex, and renal function (Figure 3). The adjusted mean difference of the preoperative levels of NT-proANP between patients with and without postoperative delirium was 0.13 ± 0.41 pmol/L (effect size: d = 0.08; unadjusted: 0.18 ± 0.43, d = 0.10). For NT-proCNP, the adjusted mean difference was 0.90 ± 0.33 pmol/L (effect size: d = 0.644; unadjusted: 0.92 ± 0.34, d = 0.63). Later in the individuals, natriuretic peptides did not show decisive results (Supplementary Table S1).

**Figure 4:**
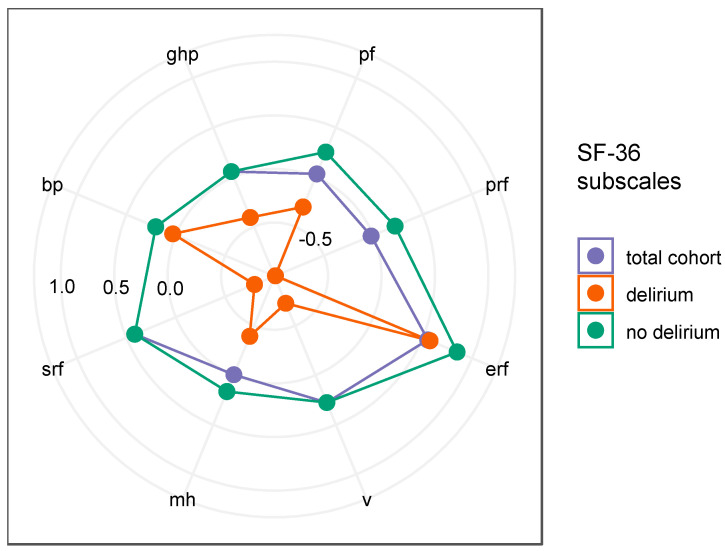
Depicts the results of the preoperative Short Form 36 Health Survey Questionnaire (SF-36), where 1.0 corresponds to a change in one standard deviation (SD). For example, physical role functioning (prf) was preoperatively reduced by one SD in patients with later delirium. Blue dots: whole cohort. Red dots: patients with occurrence of delirium. Green dots: no occurrence of delirium. The subcategories of the SF-36 are physical functioning (pf), emotional role functioning (erf), vitality (v), mental health or emotional wellbeing (mh), social role functioning (srf), bodily pain (bp), and general health perceptions (ghps), and are described in figures.

Evaluation of the SF-36 questionnaire, which was already performed preoperatively, showed significant differences between the individual characteristics. Significant differences in physical role functioning (prf) (*p* = 0.036), vitality (v) (*p* = 0.004), and social role functioning (srf) (*p* = 0.008) in the subcategories of the SF-36 were already found preoperatively in patients with later delirium (Figure 4, Supplementary Table S2).

A detailed analysis of anesthetic parameters and vital signs was performed. Although the analysis did not reveal a significant difference in the duration of surgery nor anesthesia, the data suggest that a longer duration of the procedure (mean 40 min) is associated with subsequent delirium. It is noticeable that patients with postoperative delirium cumulatively had a higher amount of propofol administered (*p* = 0.020), but not an increased alveolar concentration of sevoflurane (Table 2). A longer duration of surgery is associated with the great complexity of the procedure. The aortic clamping time, reperfusion time, and duration of the total CPB were compared. No relevant differences were found (Table 2).

## 4. Discussion

In this prospective observational study, we investigated the effects of natriuretic peptide levels drawn from peripheral blood on the development of delirium after cardiac surgery under CPB. The mean preoperative levels of NT-proCNP, but not NT-proANP, were found to be significantly associated with delirium even before the surgical intervention. Natriuretic peptides might support risk assessment as well as the early detection of postoperative delirium [13,25]. From previous work, we knew that levels of biomarkers for delirium are found to be specific after surgery [13]. This is why, as a secondary hypothesis, we wanted to examine the postoperative levels of natriuretic peptides. In contrast to our secondary hypothesis, NT-proANP was not associated with delirium. As ANP is predominantly released by cardiomyocytes like BNP, the results of the measurement of ANP and BNP may be confounded by the surgical procedure. In contrast to other studies that proposed biomarkers for preoperative assessment from postoperative drawn blood, our approach was contrary. With the non-significant results of the NTproCNP levels after surgery compared to the significant results of the NTproCNP levels before surgery, we were able to show that NTproCNP is a specific preoperative biomarker for delirium risk before surgery.

The preoperative identification of a structured risk profile for the occurrence of postoperative delirium can help optimize individual patient care. For example, a patient with an unremarkable history and low risk for postoperative delirium may require less personal care than a patient at high risk for postoperative delirium. In this case, non-pharmacological prophylaxis and screening for postoperative delirium should be performed. Thereby, especially the management of unresponsive patients after cardiac surgery prone to hypoactive delirium may be improved.

The potential new biomarker NT-proCNP may thus play a future role in the management of delirium in cardiac surgery. As the most prevalent natriuretic peptide in the brain, CNP plays a crucial physiological role in the CNS and for the integrity of the blood–brain barrier. However, a wider contribution of the peptide to the physiological regulation of cognition, motor function, and the integrity of the blood–brain barrier has not been fully elucidated, despite previous work hinting at a role for CNP in these settings [38]. Anxiogenic features may play an additional role but have contradictory results in the literature [38]. In the light of these works on CNP, we are not able to isolate a possible role of NT-proCNP as a surrogate for neuronal dysfunction, a marker for blood–brain barrier dysfunction, due to its high concentration in the brain or even as an agent involved in the pathophysiology with delirium. Nevertheless, higher levels of NT-proCNP indicate a release from the brain that might relate to a greater sensitivity for inflammatory stimuli induced by cardiac surgery. There are no therapeutic approaches for this fact. As the origin of increased NTproCNP is unclear, future studies may elucidate this and propose distinct cutoff values as well as confounding factors for a pathological concentration of NTproCNP. Admittedly, there are well-studied methods for evaluating blood–brain barrier dysfunction. Usually, these require a lumbal puncture to acquire cerebrospinal fluid, contradicting cardiac surgery because of the risk of spinal hematomas. In conclusion, albeit NT-proCNP features only a sheer approximation of the function of the blood–brain barrier, it allows an insight exceeding that of unspecific biomarkers of inflammation or neuronal damage. Lastly, this work is an exploratory approach that must be confirmed by further clinical and translational studies.

We found patients who already suffer from abnormalities in physical role functioning, vitality, and social functioning to be at high risk for developing delirium. In addition to a detailed medical history (especially gender, age, previous illnesses, long-term medication, and alcohol consumption), standardized questionnaires such as the Beck Depression Inventory II or the Short-Form 36 are very suitable for recording the risk profile, as they have been validated and used in various areas of clinical studies for years. This fact underlines that an approach to improve the preoperative collection of these data should be considered.

It is known that a stable mental, physical, and psychosocial state plays an important role in the rapid recovery of sick patients and thus, may influence future concepts such as cognitive and psychological prehabilitation before elective surgery.

Longer surgery and use of CPB are known to be strong risk factors for delirium in cardiac surgery. The duration of surgery was on average 40 min longer in patients with later delirium, usually reflecting the complexity of the procedure. Consequently, a cumulatively higher total dose of propofol results from longer surgery. However, the CPB time, aortic clamping time, reperfusion time, and duration of heart–lung machine use did not differ significantly between the two groups. Thus, we conclude that intraoperative complications were not likely the cause of delirium. However, NTproCNP value predicted delirium independent of these factors before surgery. Our interpretation is that when NTproCNP is elevated, the surgeon should aim for a less prolonged and complicated operation. Of course, this hypothesis should first be investigated in a prospective study.

Combined aortic surgery, endocarditis procedures, or procedures with hypothermic cardiac arrest overshadow the effects of more subtle risk factors for delirium, which is why these procedures were excluded from the outset. In addition, endocarditis carries an increased risk of cerebral embolism, which must be considered as a differential diagnosis for delirium in such cases [39].

Recognition of delirium is the critical step in its treatment because it is known that delirium is often misdiagnosed, detected too late, or not detected at all in up to 50% of cases. In particular, hypoactive delirium is difficult to recognize. The earlier therapeutic measures are taken, the better the outcome for the patient will be. It is known that minimizing stress, for example, by creating a familiar environment, can significantly help alleviate symptoms and provide appropriate treatment for postoperative delirium. A preoperative risk prediction using biomarkers like NT-proCNP might help to improve care for the prevention and therapy of delirium as well as guide caregivers to accurately search for symptoms of delirium, especially in hypoactive patients after cardiac surgery.

Strengths and weaknesses can be identified for our study. One strength of the study is a representative high number of cases. There was a certain homogeneity in the study population. While we acknowledge that the selected population may not fully represent the real-life cohort, our intention was to create a controlled environment to isolate the specific effects under investigation. This approach allows for a clearer understanding of the variables in question, which can be more challenging in a more heterogeneous group.

Regarding the inclusion criteria, we aimed to include participants who met specific health and demographic parameters, as captured by our inclusion criteria, to reduce confounding variables and increase the internal validity of our results. We recognize that this approach may limit the generalizability of our findings. However, it was necessary to ensure that the primary outcomes could be accurately measured without the interference of additional risk factors.

For future studies, we plan to include a more diverse cohort, including higher-risk patients, to enhance the applicability of our results to a broader population. This will help address the current study’s limitations and provide a more comprehensive understanding of the effects across different patient groups.

Primary and secondary endpoints were clearly defined and could be mapped. Standardized test procedures were used, which are already established in practice. On the other hand, there are large fluctuations in the interventions performed, especially in the duration of the surgery, which certainly has an influence on the outcome. There is also a high rate of screening failure in our patient collective. Only 24% of the screened patients initially fulfilled the inclusion criteria. However, 16% fulfilled exclusion criteria during the ongoing study, so that in the end only 19% of the screened patients provided data to our cohort, limiting the generalizability of our results.

The relatively small sample size of 80 patients limits the statistical power and generalizability of our findings. While these findings suggest that preoperative NT-proCNP values may help to identify patients at higher risk of delirium, the moderate specificity suggests a risk of false positive results. Furthermore, the absence of an external validation cohort restricts reproducibility across different populations. Further validation in larger, multi-center cohorts is necessary to refine the prediction thresholds and improve the clinical applicability of the biomarker. Besides that, the inclusion of NT-proCNP in multivariable prediction models alongside established delirium risk factors could increase its overall utility in clinical practice. Future multicenter studies are needed to confirm our findings, establish predictive thresholds, and evaluate the clinical applicability of NT-proCNP in stratifying postoperative delirium risk. Confounding factors must also be taken into account and included in the interpretation of the results. The analysis of anesthesiological parameters during the operation (amount/type of medication, duration of anesthesia, vital signs, blood loss, blood substitutes) showed no significant difference. However, there are also confounding factors that cannot be adequately filtered out. Emotional and psychological stress can lead to a lack of concentration, and general mood swings in a critical life situation can influence the motivation to participate in the study. Physical functionality and vitality also have an influence on general and therefore also cognitive performance. Here, the SF-36 shows us results that support this thesis (Figure 4). Although we tried to identify participants at risk of depression in advance using the Beck Depression Inventory-II, changes in mental well-being are not uncommon.

A regular and standardized assessment of delirium should take place in ICUs, as patients with delirium after standard procedures in cardiac surgery experience long-lasting self-reported deterioration in their physical role, vitality, and social function.

In addition, preoperative risk stratification using standardized questionnaires and medical history should always be performed to ensure optimal medical care. Patients with a high-risk profile in particular could benefit from close monitoring and care by trained personnel. Care could be taken to ensure that high-risk patients do not receive medications that favor the development of POD (e.g., anticholinergics). In addition, specific care would be possible to minimize anxiety and stress. This would also optimize the implementation of structured procedures in an unfamiliar environment. Preoperative biomarkers such as NTproCNP can further improve this approach and enable more precise risk stratification.

## 5. Conclusions

The natriuretic peptide NT-proCNP could prove to be a predictive marker to identify patients prone to delirium in cardiac surgery. A functional assessment might especially refine the significance of this biomarker. Patients with this risk profile could specifically profit from individual preventive measures and close-meshed delirium screening and subsequent therapy.

However, we would like to emphasize that our results should be interpreted as hypothesis generating rather than definitive. This should be re-evaluated and confirmed in larger validation studies using multivariable risk models.

## Figures and Tables

**Table 1:** Characteristics of the study participants.

	Total (N = 80)	Delirium (N = 28)	No Delirium (N = 52)	*p*
Sex [female] ^1^	19/80 (24%)	7/28 (25%)	12/52 (23%)	0.781
Age [years] ^2^	72 (66–76)	74 (68–77)	70 (65–76)	0.332
Weight [kg] ^2^	85 (±2)	85 (±3)	84 (±2)	0.681
Height [cm] ^2^	172 (167–178)	172 (167–178)	172 (167–178)	0.964
Education [years] ^2^	9 (8–10)	9 (8–13)	9 (8–10)	0.403
Preoperative Hb [g/dL] ^2^	13.3 (12.0–13.9)	12.9 (11.6–13.8)	13.4 (12.4–13.9)	0.388
ADL ^2^	100 (95–100)	100 (100–100)	100 (90–100)	0.406
BDI ^2^	0 (0–0)	0 (0–0)	0 (0–0)	0.212
MMSE	28 (27–29)	28 (27–29)	28 (27–29)	0.813
EF%	60 (60–60)	60 (55–60)	60 (60–60)	0.236
ADS	0 (0–0)	0 (0–0)	0 (0–0)	0.574
**SF-36 subcategories**				
Physical functioning ^1^	0.03 (±0.14)	−0.30 (±7.21)	0.25 (±0.16)	0.077
Physical role functioning ^2^	−0.02 (−0.99–0.82)	−0.99 (−0.99–0.12)	0.22 (−0.99–1.01)	0.036
Emotional role functioning ^2^	0.55 (−1.33–0.85)	0.06 (−1.53–0.85)	0.85 (−0.74–0.85)	0.674
Vitality	0.28 (−0.73–0.78)	−0.73 (−1.23–0.28)	0.28 (−0.2–1.03)	0.004
Mental health or emotional wellbeing ^1^	−0.01 (±0.14)	−0.39 (±0.21)	0.16 (±0.18)	0.053
Social role functioning ^2^	0.41 (−0.80–1.02)	−0.80 (−1.40–0.41)	0.41 (−0.46–1.02)	0.008
Bodily pain ^2^	0.20 (−0.64–0.98)	0.03 (−1.18–0.98)	0.20 (−0.57–0.98)	0.635
General health perceptions ^2^	0.056 (−0.56–0.67)	−0.41 (−0.87–0.29)	0.06 (−0.25–0.98)	0.054

^1^ N (%) and Chi^2^-test, ^2^ Median (25th–75th Quantile) and Wilcoxon test. Hb: hemoglobin, ADL: Activities of Daily Living, BDI: Beck Depression Inventory-II, MMSE: Mini-Mental Status Examination, EF%: ejection fraction, ADS: anticholinergic drug scale, SF-36: Short Form 36 Health Survey Questionnaire.

**Table 2:** Results of intraoperative data.

	Total (N = 80)	Delirium (N = 28)	No Delirium (N = 52)	*p*
surgery time (min) ^2^	306 (266–376)	344 (261–436)	297 (269–341)	0.148
propofol dose (mg per kg body weight^−1^ h^−1^) ^2^	5.34 (4.67–5.87)	5.44 (4.27–5.99)	5.32 (4.71–5.83)	0.880
propofol, cumulative dose (mg) ^1^	2413 (±71)	2635 (±148)	2293 (±69)	0.020
sufentanyl, total (µg) ^2^	463 (372–593)	500 (343–654)	457 (380–556)	0.664
sevoflurane (MAC) ^2^	0.7 (0.5–1.1)	0.9 (0.5–1.6)	0.65 (0.5–1.0)	0.126
crystalloid infusion (mL) ^1^	2624 (±794)	2800 (±151)	2530 (±109)	0.148
colloid infusion (mL) ^2^	425 (100–500)	150 (100–463)	500 (100–500)	0.226
blood loss (mL) ^2^	500 (488–1000)	600 (488–1000)	500 (425–1000)	0.177
autologous blood re-transfusion (mL) ^2^	263 (200–350)	300 (230–370)	250 (200–340)	0.147
platelet concentrates (mL) ^2^	300 (300–600)	300 (300–600)	300 (300–400)	0.411
red packed cell concentrates (mL) ^2^	250 (250–675)	500 (250–600)	250 (250–750)	0.835
fresh frozen plasma (mL) ^2^	750 (750–750)	750 (750–750)	750 (625–112)	0.923
hydrocortisone (mg) ^2^	124 (100–145)	125 (100–157)	113 (100–144)	0.696
maximum MAP (mmHg) ^1^	104 (±19)	99 (±4)	107 (±2)	0.063
minimum MAP (mmHg) ^2^	44 (36–51)	102 (86–113)	44 (36–51)	0.864

^1^ Chi^2^-test, ^2^ Median (25th–75th Quantile), MAC: minimum alveolar concentration, MAP: mean arterial pressure.

## Data Availability

The original contributions presented in this study are included in the article/Supplementary Materials. Further inquiries can be directed to the corresponding author.

## Supplementary Materials

The following supporting information can be downloaded at: https://www.mdpi.com/article/10.3390/jcm14051533/s1, Figure S1: ROC analysis; Table S1: Results of Natriuretic Peptides Data at Secondary Endpoints; Table S2: Differences in the surgical procedures.
