# Supplementary material for: Natriuretic Peptides as a Predictor for Delirium After Cardiac Surgery: A Prospective Observational Study
Source: J Clin Med. 2025 Feb 25;14(5):1533. doi: 10.3390/jcm14051533 (PMC11900125; doi:10.3390/jcm14051533)

Supplementary Tables

Supplementary Table S1: Results of Natriuretic Peptides Data at Secondary Endpoints

	**Total** (N = 80)[1]	**Delirium** (N = 28)[2]	**No Delirium** (N = 52)[2]	**P**
NT-proCNP (pmol/L) 1st postoperative day	4.70 (2.7)	5.25 ± 0.48	4.40 ± 0.38	0.183
NT-proCNP (pmol/L) 2nd postoperative day	6.47 (4.0)	6.09 ± 0.73	6.48 ± 0.58	0.658
NT-proCNP (pmol/L) 3rd postoperative day	5.11 (3.0)	4.82 ± 0.55	5.08 ± 0.42	0.693
proANP (pmol/L) 1st postoperative day	5.33 (3.0)	5.48 ± 0.58	5.20 ± 0.45	0.676
proANP (pmol/L) dismissal	4.79 (2.2)	4.62 ± 0.50	4.51 ± 0.39	0.849
[1] Mean (SD), [2] Least square mean estimates ± standard error adjusted for sex, age, and renal function.				

Supplementary Table S2: Differences in the surgical procedures

	**Total** (N = 80)	**Delirium** (N = 28)	**No Delirium** (N = 52)	**P**
**any CABG surgery**	**46**	16 (34.8 %)	30	>0.999
only CABG surgery<br>Number of vessels, - 1<br>- 2 vessel<br>- 3 vessel<br>- 4 vessel<br>- 5 vessel	35<br>2<br>6<br>15<br>10<br>2			
**only valve replacement**	**34**	12 (35,3%)	22	>0.999
**any valve replacement**	**45**			
aortic valve<br>- replacement<br>- + MKR<br>- + TKR<br>- + CABG	31<br>23<br>1<br>2<br>5			
mitral valve<br>- replacement<br>- reconstruction<br>- + CABG	14<br>6<br>3<br>5			
**CABG** coronary artery bypass graft, **MKR** mitral valve reconstruction, **TKR** tricuspid valve reconstruction				

Supplementary Figure S1: ROC analysis

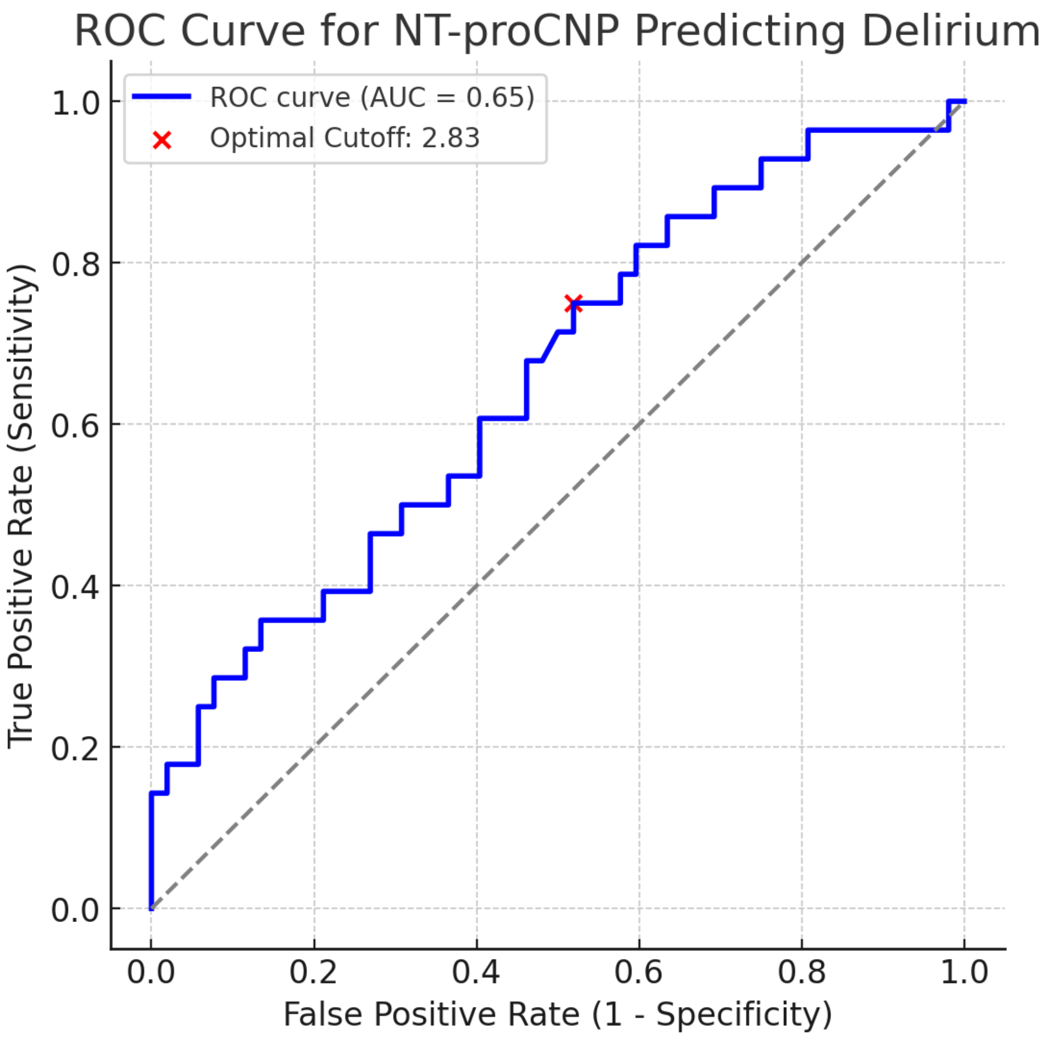

Supplement: Supplementary file 1 [file jcm-14-01533-s001.zip › jcm-3461006-supplementary.pdf]
